# Impact of multiple food environments on body mass index

**DOI:** 10.1371/journal.pone.0219365

**Published:** 2019-08-07

**Authors:** Adriana Dornelles

**Affiliations:** Department of Economics, Arizona State University, Tempe, AZ, United States of America; Cincinnati Children's, UNITED STATES

## Abstract

**Background:**

Although the relationship between residential food environments and health outcomes have been extensively studied, the relationship between body mass index (BMI) and multiple food environments have not been fully explored. We examined the relationship between characteristics of three distinct food environments and BMI among elementary school employees in the metropolitan area of New Orleans, LA. We assessed the food environments around the residential and worksite neighborhoods and the commuting corridors.

**Research methodology/principal findings:**

This study combined data from three different sources: individual and worksite data (ACTION), food retailer database (Dunn and Bradstreet), and the U.S. Census TIGER/Line Files. Spatial and hierarchical analyses were performed to explore the impact of predictors at the individual and environmental levels on BMI. When the three food environments were combined, the number of supermarkets and the number of grocery stores at residential food environment had a significant association with BMI (β = 0.56 and β = 0.24, p < 0.01), whereas the number of full-service restaurants showed an inverse relationship with BMI (β = -0.15, p < 0.001). For the commute corridor food environment, it was found that each additional fast-food restaurant in a vicinity of one kilometer traveled contributed to a higher BMI (β = 0.80, p <0.05), while adjusting for other factors. No statistical associations were found between BMI and worksite food environment.

**Conclusions:**

The current study was the first to examine the relationship between BMI and food environments around residential neighborhoods, work neighborhoods, and the commuting corridor. Significant results were found between BMI and the availability of food stores around residential neighborhoods and the commuting corridor, adjusted for individual-level factors. This study expands the analysis beyond residential neighborhoods, illustrating the importance of multiple environmental factors in relation to BMI.

## Introduction

Obesity is a national health concern that has reached epidemic proportions [1–3]. There is increased evidence that the environment promotes overeating, physical inactivity, and energy imbalance [4, 5]. Numerous studies have examined the relationship between the availability of food outlets in adults’ neighborhoods and their weight [6–10]; however, findings related to this relationship are not always consistent. While some studies have found associations between body mass index (BMI) and proximity to fast-food restaurants, grocery stores, full-service restaurants, and supermarkets, others have found no relationship or produced mixed results [11]. The few longitudinal studies investigating the association between proximity to food establishments and BMI have shown significant results when fast-food restaurants and grocery stores are observed [7, 12–14].

Although there is a growing body of evidence to show that the distribution of the food retail environment may affect individual lifestyle choices, there is a limitation common to these studies and that is they have focused only on the residential neighborhood environment [15–18] (urban and/or rural areas). To date, the few studies that have investigated the relationship between weight and food outlets near homes, worksites, and schools have shown discordant findings [19–21]. In addition, studies focusing solely on worksites have focused on intervention programs within the worksite itself to promote obesity reduction rather than on accounting for the contextual factors surrounding workplace [22–27].

As most individuals encounter several environments during their daily lives, i.e. where they live, work, and/or go to school, it is important to consider the food options within these environments to more fully understand the environmental influences on people’s weight. So far, only one study has accounted for both residential and non-residential food environments; food exposure in non-residential activity places was strongly associated with overweight for men, but not women when compared to residential-only measures of exposure [28].

Thus, the present study was designed to investigate the association between BMI and the food environments in people’s neighborhoods, at their work locations, and along their commuting corridors. More specifically, we targeted our analysis on elementary school employees; therefore, expanding on previous analyses that have focused on only one food environment at a time while we consider multiple food environments.

This study is among the first to account for multiple food environments, and the development of a food environment based on participants’ commuting is a novel approach. Most of the previous studies working on the influence of the food environment on BMI have been restricted to residential neighborhoods. To date, no study has specifically considered the influence of the different types of food retailers around the worksite or along the commute route. The focus on the food environment of elementary school personnel is unique relative to the context of workplaces. A majority of previous studies performed at schools were focused on students rather than school employees. We had objective measures for height and weight and physical activity thus, eliminating biases associated with self-reporting. In addition, the food environments developed for this study were based on participants’ home and work addressees, while most previous studies gathered residential information by census tract level, ZIP codes, or block groups [6, 10, 29–31].

## Methods

### Study design

This is a cross-sectional secondary analysis of the data collected from ACTION!, a worksite intervention program for elementary school personnel. Protocols were approved by the Tulane University Institutional Review Board and a voluntary written consent was obtained from participants.

### Study sample

This study combines data from three different sources: individual and worksite data (ACTION!), a food retailer database (Dunn and Bradstreet), and the U.S. Census TIGER/Line Files (S1 File).

1. Individual and Worksite data:
  Data from ACTION Worksite Wellness for Elementary School Personnel (ACTION!) provided information at the individual and worksite level. ACTION! was a school-based worksite wellness intervention trial in a suburban school district within the Greater New Orleans area. Details on recruitment, study design and the main results of the study have been reported previously [24, 5]. In brief, it was a group-randomized trial in which the school was the unit of randomization as well as the unit of analysis. All baseline measurements were collected in the fall of 2006, and follow-up measurements in 2008. Due to the major effects of Hurricane Katrina in 2004, we decided to use the completed data from 2008, which included 866 employees nested in 22 different schools. Schools were the primary units for this trial, and the participants or secondary units were the school employees.
  Individual socio-demographic data were obtained through questionnaires at the study entry such as sex, age, gender, race/ethnicity, education level and marital status. Job category and the addresses of school employees were obtained from employee rosters provided by the schools. The worksite data, defined by the participating elementary schools, included information regarding the existence of vending machines, cafeteria, gym, playing field, and walking paths.
2. Mapping of Retail Outlets
  The locations of food outlets were obtained from the Dunn and Bradstreet (D&B) commercial database. The database included all food retailers open for business in Louisiana in 2008 with respective addresses and geographic coordinates (latitude and longitude). Information on the total number of food retailers was available at the 8-digit SIC (Standard Industrial Classification) code level [32], allowing us to separately examine the food business by type. Four types of food retailers were considered for this analysis: (1) supermarkets, (2) grocery stores, (3) full-service restaurants and (4) fast-food restaurants. This method of food store classification is consistent with previous analyses in this field [30, 33–36]. All food business addresses provided by D&B were doubled-checked using the Yellow Pages and Google Maps [37, 38].
  The home and worksite address of participants residing in three parishes—Orleans, Jefferson, and St. Charles—of the greater New Orleans area were geocoded to longitude and latitude coordinates and matched to census tracts in New Orleans using ArcGIS 9.3 (ESRI, Redlands, CA). We created a 1-km buffer in all directions from the center point of each home and worksite and identified relevant food businesses within the buffer. We then defined the *home food environment* by the count of all supermarkets, grocery stores, full-service restaurants, and fast-food restaurants within the buffer. The *worksite food environment* was created by the same method described above using the address of the schools.
  To create the *commuting food environment*, we first designed a single commute route for each participant followed by the selection of food places along those routes. We used ArcGIS Network Analyst to simulate the shortest distance traveled (in kilometers) between participant’s home address (origin) and worksite location (destination). After designing each employee’s commute route, a commuting corridor was created by the area encompassing a 1-km buffer from all points along their itinerary. All food retailers within the commuting corridor were selected, classified and counted. Density measures were created by summing the quantity of each type of food retailer and dividing the sum by the total length of the commute (km).
3. Socio-environmental measures
  Data concerning the three built environments came from the 2010 U.S. Census Bureau and TIGER/Line Shape files. TIGER is an acronym for the Topologically Integrated Geographic Encoding and Referencing system which was created by the Census Bureau. The combination of U.S. Census and TIGER data gave information about street connectivity, land use mix, residential density, intersection density, and block size. The median annual household income data was extracted from the 2010 U.S. Census and it was used as an indicator of the environmental socio-economic position.

**Fig 1:**
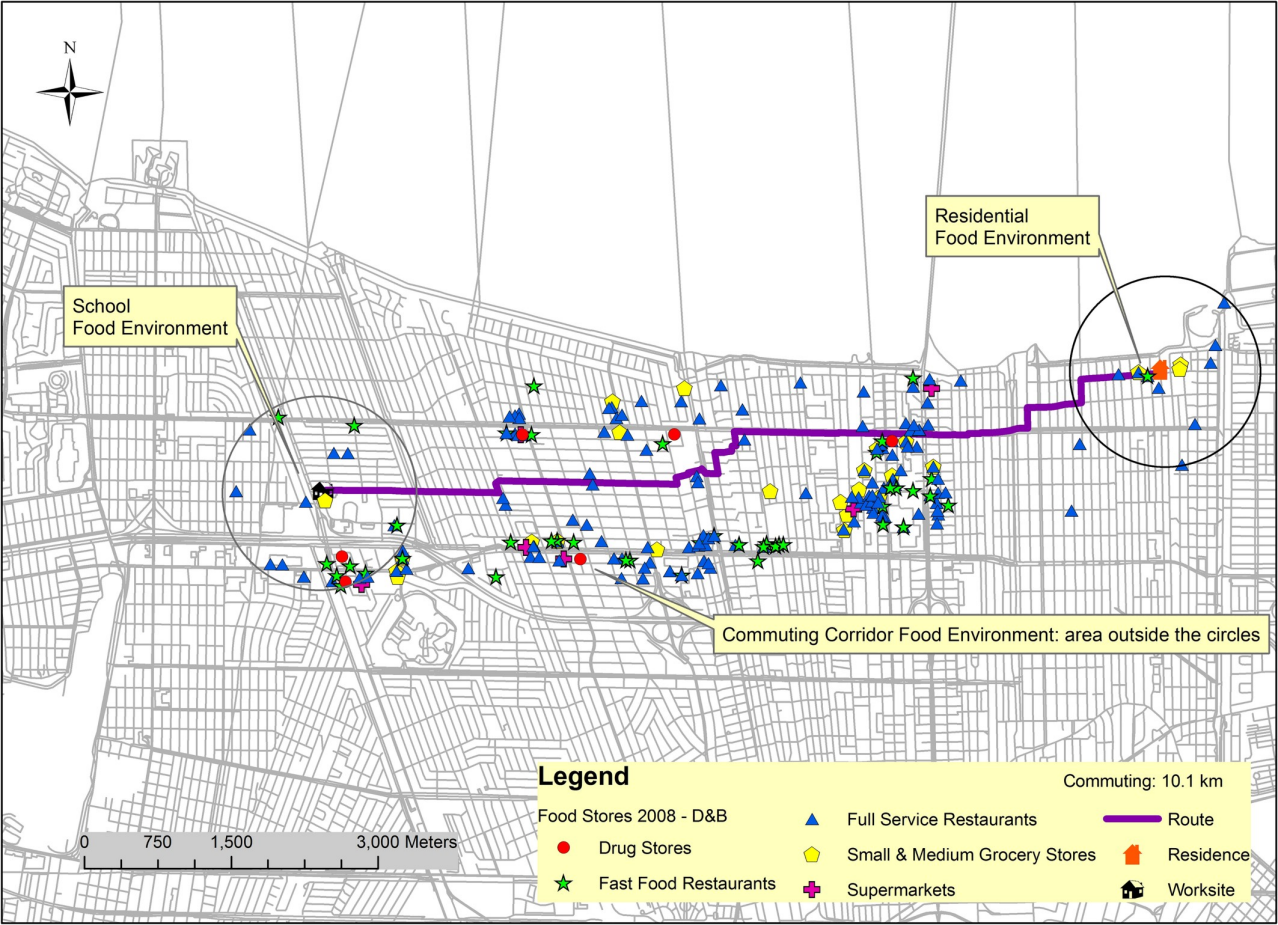
Map of food establishment locations in all 3 food environments for a fictitious participant in the greater New Orleans, LA region in 2008.

Fig 1 shows the three food environments of a hypothetical participant who lives and works in the New Orleans metropolitan area. To protect participants’ confidentially, we created this fictitious participant as an illustration of residential, worksite, and commute food environments.

### Key variables

Our primary variable of interest is BMI, calculated as weight (kg)/height (m^2^). Height and weight were measured in duplicate by trained examiners during a physical examination. Height was measured to the nearest 0.1 cm using a portable stadiometer and weight was measured to the nearest 0.1 kg with a calibrated scale. These measurements were repeated if the difference between weights and heights were ≥ 0.5 kg and ≥1 cm, respectively. Heights and weights were converted into BMI score [39]. BMI was used to classify participants as normal weight (BMI < 25), overweight (BMI 25 to 29.9), or obese (BMI ≥ 30).

*Physical activity* was obtained in 2008 by an ActiGraph uniaxial accelerometer (ActiGraph LLC, Pensacola, FL) that participants wore for 7 days except during sleep or water activities. Participants were categorized as active if they engaged in more than 30 minutes of light to moderate physical activity per day and not active, otherwise.

Other variables obtained included age (≤39 years, 40–59 years, and 60+ years), race/ethnicity (White Caucasian, African American, and other), physical activity, job category (instructional and noninstructional), household median income by census tracts, daily distance traveled, average commute time, and food stores.

### Statistical models

The data collected in this study are correlated due to its hierarchical structure. In parametric modeling, accounting for correlations at different levels increases the complexity. These models may also yield results that lead to the same conclusions as simpler models. However, we obtained a measure of intraclass correlation (ICC) at each stage and found that the intraclass correlation was large enough (ICC = 0.18) to require the use of a two-level nested structure account for dependencies in hierarchical models. These guidelines follow the rule of thumb to assist researchers faced with the challenge of choosing an appropriately complex model when analyzing hierarchical data.

Several hierarchical models were fitted for both residential and school food environments. At the residential level, participants were clustered within zip codes whereas, at the worksite level, teachers were nested within schools. Thus, hierarchical regression models were performed to determine the association between BMI, socio-demographic characteristics and food environments for residential, worksite and commuting corridor.

Let Y_ij_ denote the BMI for participant i nested in zip code j with subject’s predictors X_1jk_ denoting supermarket, X_2jk_ denoting grocery stores, X_3jk_ denoting fast food, X_4jk_ denoting restaurants, X_5jk_ denoting ethnicity, X_6jk_ denoting occupation, X_7jk_ denoting physical activity, and X_8jk_ denoting median income.

Model 1 addresses the association of residential food environment and BMI with the superscript R denoting the residential food environment as:

$$Y_{ij}=\beta_{0}+\beta_{1}X_{1jk}^{R}+\beta_{2}X_{2jk}^{R}+\beta_{3}X_{3jk}^{R}+\beta_{4}X_{4jk}^{R}+\beta_{5}X_{5jk}^{R}+\beta_{6}X_{6jk}^{R}+\beta_{7}X_{7jk}^{R}+\beta_{8}X_{8jk}^{R}+\zeta_{j}+\epsilon_{ij,}$$

*ζ*_*j*_ is the random effects denoting the variation among zip code, and *ϵ*_*ij*_ is the error term where $\zeta_{j}∼ℵ(0,\sigma_{j}^{2})$ and $\epsilon_{ij}∼ℵ(0,\sigma_{e}^{2})$

Model 2 measures the association between worksite food environment [superscript w] and BMI. The model for the *Y*_*ij*_ the BMI of participant *i* within zip codes *j*, is specified using predictors as:

$$Y_{ij}=\beta_{0}+\beta_{1}X_{1jk}^{W}+\beta_{2}X_{2jk}^{W}+\beta_{3}X_{3jk}^{W}+\beta_{4}X_{4jk}^{W}+\beta_{5}X_{5jk}^{W}+\beta_{6}X_{6jk}^{W}+\beta_{7}X_{7jk}^{W}+\beta_{8}X_{8jk}^{W}+\zeta_{j}+\epsilon_{ij,}$$

*ζ*_*j*_ is the random effects denoting the variation among schools, and *ϵ*_*ij*_ is the error term where $\zeta_{j}∼ℵ(0,\sigma_{j}^{2})$ and $\epsilon_{ij}∼ℵ(0,\sigma_{e}^{2})$

Model 3 addresses the association of commute food environment [superscript C] and BMI. The model for the *Y*_*ij*_ the BMI of participant *i* within zip codes *j*, is specified using predictors as:

$$Y_{ij}=\beta_{0}+\beta_{1}X_{1jk}^{C}+\beta_{2}X_{2jk}^{C}+\beta_{3}X_{3jk}^{C}+\beta_{4}X_{4jk}^{C}+\beta_{5}X_{5jk}^{C}+\beta_{6}X_{6jk}^{C}+\beta_{7}X_{7jk}^{C}+\beta_{8}X_{8jk}^{C}+\zeta_{j}+\epsilon_{ij,}$$

*ζ*_*j*_ is the random effects denoting the variation among zip code, and *ϵ*_*ij*_ is the error term where $\zeta_{j}∼ℵ(0,\sigma_{j}^{2})$ and $\epsilon_{ij}∼ℵ(0,\sigma_{e}^{2})$

Model 4 is a compilation of variables from models 1, 2 and 3.

Model 4 addresses jointly the association of residential, worksite, commute food environments and BMI. The model for the *Y*_*ij*_ the BMI of participant *i* within zip codes *j*, is specified using predictors as: $Y_{ij}=\beta_{0}+\beta_{1}X_{1jk}^{R}+\beta_{2}X_{2jk}^{R}+\beta_{3}X_{3jk}^{R}+\beta_{4}X_{4jk}^{R}+\beta_{5}X_{1jk}^{W}+\beta_{6}X_{1jk}^{W}+\beta_{7}X_{2jk}^{W}+\beta_{8}X_{3jk}^{W}+\beta_{9}X_{1jk}^{C}+\beta_{10}X_{2jk}^{C}+\beta_{11}X_{3jk}^{C}+\beta_{12}X_{4jk}^{C}+\beta_{13}X_{5jk}+\beta_{14}X_{6jk}+\beta_{15}X_{7jk}+\beta_{16}X_{8jk}+\zeta_{j}+\epsilon_{ij,}$

*ζ*_*j*_ is the random effects denoting the variation among zip codes, and *ϵ*_*ij*_ is the error term where $\zeta_{j}∼ℵ(0,\sigma_{j}^{2})$ and $\epsilon_{ij}∼ℵ(0,\sigma_{e}^{2})$

In the fit of Models, models 1, 2, 3, and 4 were checked for the presence of multicollinearity which resulted in no significant correlation as all the variance inflation factors were below 3.5. These are normal-normal hierarchical models. The models showed that the variance of random effects were significant and thus were necessary components in the model. All analyses were performed in STATA/SE 15.1 (College Station, TX).

## Results

Of the 866 participants in the ACTION 2008 cohort, 31 participants were excluded from analysis because they were ineligible (e.g., did not provided a valid home address) (n = 2), pregnant or breastfeeding (n = 4), did not reside in Orleans, Jefferson, or St. Charles parishes (n = 3), were not working at one of the ACTION schools in 2008 (n = 2), or had a BMI greater than 50 (n = 20). The current paper focused on the three specific food environments; therefore, we also excluded 109 participants who resided less than 2 kilometers from schools because the three food environments would overlap. There were also 11 participants who had to commute more than 27 kilometers and were excluded from the analytical sample, as their inclusion would produce an excessive number of food business. Few males were interviewed in the original sample and remained after these exclusions (n = 5), thus analyses were restricted to females. Thus, the final sample size for this study contained 22 schools and a total of 710 employees.

A summary of the demographic characteristics of the survey respondents is given in Table 1. 72.7% of respondents were white, 72.8% were instructional personnel and 63.8% were 40–59 years of age. The mean BMI is 29.4 ± 6.7 kg/m^2^. The majority was classified as either obese (41.7%) or overweight (29.3%), and only 15.1% of participants were engaged in more than 30 minutes of daily physical activity. The average daily distance traveled was 18.4 ± 12.2 kilometers, and the daily commute time was 25.2 ± 2.9 minutes. The median household income was $38,852 ± $7,241.

**Table 1:** Demographic characteristics of survey respondents (n = 710).

Variable	n	Mean ± SD (%)
BMI (kg/m^2^)	710	29.4 ± 6.7
Underweight/Normal (< 25 kg/m^2^)	206	(29.0)
Overweight (25–30 kg/m^2^)	208	(29.3)
Obese (> 30 kg/m^2^)	296	(41.7)
Age (years)		
≤39	155	(21.8)
40–59	453	(63.8)
≥60	102	(14.4)
Race		
White	516	(72.7)
African American	152	(21.4)
Other	42	(5.9)
Job Category		
Instructional	517	(72.8)
Non–Instructional	193	(27.2)
Median Income (in $ 1000s)	710	38.85 ± 7.2
Daily distance traveled	710	18.4 ± 12.1
Daily traveled time	710	25.2 ± 2.9
Physical Activity		
< 30 minutes/day	603	(84.9)
≥ 30 minutes/day	107	(15.1)

Table 2 depicts the distribution of food retails by food environment. The most frequently observed food businesses in all three food environments were grocery stores followed by full-service restaurants. The average number of full-service restaurants was higher than any other food retailer type for all three food environments. Overall, the number of food retailers were similar for both residential and worksite food environments.

**Table 2:** Distribution of food retails by food environment.

Food Retail Means ± SD (IQR)	Residential	Worksite	Commute
Supermarkets	0.4 ± 0.7 (0–3)	0.5 ± 0.7 (0–1)	1.6 ± 1.7 (0–0.26)
Grocery stores	4.2 ± 3.8 (1–6)	4.6 ± 3.1 (2–6)	20.1 ± 19.8 (1.1–2.6)
Fast-food restaurants	3.9 ± 3.8 (1–6)	3.1 ± 2.4 (1–4)	19.0 ± 17.5 (1.0–2.7)
Full-service restaurants	10.8 ± 10.2 (4–14)	12.1 ± 9.5 (7–15)	54.4 ± 60.3 (1.6–2.6)

### Individual food environment analysis

Model 1 fits the effects of the residential food environment on BMI. The number of supermarkets and grocery stores located within a 1-km radius of the participants’ homes was significantly positively associated with an increase in BMI ($\hat{\beta }$ = 0.60 and $\hat{\beta }$ = 0.25, respectively *P*<0.05). Conversely, BMI decreased by 0.13 units as a new full-service restaurant was established within 1-km radius of participants’ homes.

Model 2 addressed the worksite food environment factors and found no significant associations between BMI and any type of food business while accounting for ethnicity, occupation, physical activity and median household income.

Model 3 examined the four types of food businesses located in the commute corridor food environment and BMI. After controlling for socioeconomic variables, a significant association between BMI and fast-food restaurants was identified as well as an association between BMI and full-service restaurants as well. Each additional fast-food restaurant in a vicinity of one kilometer traveled contributed to a higher BMI ($\hat{\beta }$ = 0.70, P <0.05).

The results of individual-level characteristics were similar throughout models 1 to 4, controlling for the food environment. White participants had, on average, lower BMI than all other races combined. Higher median income was negatively associated with BMI; an increase of U$1,000 will decrease, on average, school employee’s BMI by 0.07, holding everything else constant (Table 3, model 1). School personnel who were physically active (more than 30 min/day) showed lower BMI than those who exercise 30 minutes a day or less. No statistical difference in BMI was found between instructional versus non-instructional participants.

**Table 3:** Multivariable models: Predicting the impact on food environment (FE) on BMI‡ (n = 710).

Variable	Model 1	Model 2	Model 3	Model 4
	Residential	Worksite	Commute Corridor	All 3 FE
	β (SE)	β (SE)	β (SE)	β (SE)
*Residential FE*				
Supermarkets	0.60 (0.30)^b^	-	-	0.56 (0.35)^b^
Grocery stores	0.25 (0.06)^c^	-	-	0.24(0.07)^c^
Fast-food restaurants	0.05 (0.06)	-	-	0.11 (0.09)^a^
Full-service restaurants	-0.13 (0.03)^c^	-	-	-0.15 (0.03)^c^
*Worksite FE*				
Supermarkets	-	-0.27 (0.55)	-	-0.48 (0.43)
Grocery stores	-	-0.20 (0.14)	-	-0.24 (0.13)^a^
Fast-food restaurants	-	-0.10 (0.16)	-	-0.03 (0.10)
Full-service restaurants	-	0.01 (0.05)	-	0.04 (0.04)
*Commute FE†*				
Supermarkets	-	-	-1.08 (1.64)	-0.97 (1.60)
Grocery stores	-	-	0.06 (0.27)	-0.14 (0.20)
Fast-food restaurants	-	-	0.70 (0.50)^b^	0.80 (0.24)^b^
Full-service restaurants	-	-	- 0.69 (0.47)	-0.61 (0.44)
*Individual Level§*				
Instructional	0.52 (0.65)	0.32 (0.57)	0.50 (0.57)	0.60 (0.64)
White	-1.47 (0.55)^b^	-1.74 (0.58)^b^	-1.68 (0.58)^b^	-1.35 (0.58)^b^
Physical Active	-1.80 (0.60)^b^	-1.99 (0.69)^b^	-2.04 (0.69)^b^	-1.98 (0.60)^b^
Median income (in 1000s)	- 0.07 (0.04)^a^	-0.10 (0.04)^b^	-0.10 (0.04)^b^	-0.11 (0.04)^b^

^a^ p< 0.10

^b^ p< 0.05

^c^ p<0.001

‡Each model includes all type of food retailers within 1-km radius of the food environment

† Density of facilities per km travelled.

*§* Non-instructional, non-white and exercising less than 30 min/day were used as reference.

The analyses provided by Models 1, 2, and 3 gave us the opportunity to build a more comprehensive model that simultaneously addresses questions with as many covariates in consideration as shown in Model 4.

### Multiple food environment analysis

Model 4 includes the three food environments (FE): residential, worksite, and commute corridor. This affords us the opportunity to simultaneously assess the impact on the participants’ BMI. Controlling for worksite, commute corridor food environments, and socio-demographic characteristics, the number of supermarkets and the number of grocery stores had significant association with BMI ($\hat{\beta }$ = 0.56 and $\hat{\beta }$ = 0.24, P < 0.01), while the number of full-service restaurants showed an inverse relationship with BMI ($\hat{\beta }$ = -0.15, P < 0.001).

We found no association between food retailers within a 1-km radius of the worksite and BMI, while accounting for residential and commute corridor food environments. The number of fast-food restaurants located along the commute corridor was positively associated with BMI in the full model. This is consistent with our findings in Model 3, ($\hat{\beta }$ = 0.80 vs. $\hat{\beta }$ = 0.70).

Overall, with everything under consideration, we found that number of supermarkets, grocery stores, fast food restaurants in the residential FE, and the fast-food restaurants in the Commute FE, had an increased but significant effect on BMI while the number of full-service restaurants at worksite FE, and the number of grocery stores at worksite FE had a decreased but significant effect on BMI. The full model only disagreed in the predictors we considered significant are those in the p-value range (0.05–0.10). In such cases, we would probably rely on the full model.

## Discussion

This study assessed the relationship between elementary school employees’ BMI and multiple food environments, taking into account different types of food retailers. We examined whether food service retailers have an impact on BMI while addressing both single and multiple food environments. We limit our discussion to findings obtained from Model 4 because of its importance in measuring multiple food environments.

### Factors associated with BMI and the residential food environment

The results showed that the number of supermarkets was associated with an increase in BMI which is opposite to what we have theorized. While some studies reported comparable results [10, 40, 41], the majority of the studies reported either negative associations [29, 42–44] or null findings [34, 45, 46]. The number of grocery stores located in residential areas was significantly related to an increase in one’s BMI, whereas the presence of full-service restaurants showed a significant decrease in BMI. Our findings are supported by other studies [7, 10, 47, 48] in which BMI is impacted by the total number of grocery stores found in residential neighborhoods. Several studies have shown a negative association with BMI [7, 8, 48, 49] and the presence of full-service restaurants in residential neighborhoods, in accordance with our findings. However, unlike our analysis, other studies were not able to report a significant association between BMI and the number of full-service restaurants [7, 9, 48]. We did not find any association between BMI and the number of fast-food restaurants in the residential food environment. Other studies that examined a similar relationship reported the same outcome [19, 50–53].

### Factors associated with BMI and the worksite food environment

In the case of worksite food environment, we observed no significant associations between BMI and the number of food businesses. Prior research found no association between the density of food outlets (restaurants and grocery stores) and BMI among elementary school children [19, 52]. Few researchers were able to find an association between fast-food consumption, energy intake, and diet quality [8, 34, 54–55] Other studies that focused on the proximity of fast-food restaurants to schools showed that the majority of fast-food establishments are more concentrated around public schools than private schools [56–58]. However, to best of our knowledge, the analyses in those studies targeted students rather than school employees.

Our insignificant findings for the worksite food environment may be due to a number of factors. First, school personnel have limited time for lunch, which might restrict their access to food retailers outside the school. In addition, we did not evaluate the food environment inside schools, such as access to and the use of vending machines or how often employees eat at the onsite cafeteria. A prior study on dietary intake from ACTION revealed that most of the school employees consumed a high mean of daily calories (1,962 ± 555), and the diets of approximately 45% of employees exceeded dietary fat recommendations [25]. Thus, little is known about the food environment within and around schools, and results from this study highlight the need for further research into employees’ access to food establishments near schools, the food environment in schools, and the connection between these two factors and school employees’ weight.

### Factors associated with BMI and the commute corridor food environment

Results estimating the association between BMI and food retailers along the commute corridor were consistent for single and multiple food environments. The results indicate that the density of fast-food restaurants within 1-km of participants’ routes was positively associated with BMI. There are no known studies that have examined the association of fast-food restaurant availability and BMI along the commuting corridor. Over the past decades, consumption of away-from-home food at fast-food places has increased substantially. This situation along with fast-food portion sizes, results in the population’s overall higher consumption of fat, cholesterol, and carbohydrates and therefore increased weight and obesity [59]. In addition, incentives of price and time are particularly salient for full-time workers who often work long hours and do not have time to cook at home. According to economists, fast-food consumption is higher in relation to consumption of home-cooked foods because of time constraints [60, 61]. A recent study of adolescents and adults who eat regularly at fast-food restaurants reported three main reasons people choose fast food: it is fast, easy, and tastes good. The study also reported that those with a bachelor’s degree or higher level of education were more likely to eat fast food because they are too busy to cook food at home compared to those with less education [62]. Although novel, the commute food environment created in this study has limitations. The commute routes were hypothetical and based on short distance travel, therefore the use of highways may impact the stops for food. It was also assumed that all participants drove to schools alone (not carpooling or dropping children off at a different school), used the same home-to-work commute every day, and did not change their home or work address during the year of 2008. Overall, our assumptions on the commute routes are in accordance with transportation studies of commuting choice, suggesting that a majority of the workers choose their commute route based on the shortest distance to minimize time, and only 20% of drivers change itineraries frequently [63–66].

Interventions that targets reducing fast-food meal frequency and accessibility of fast-food restaurants should be considered. Factors to explore in future research include participant’s exact commute routes, what types of food stores they visit, and what types of food they buy in their home-to-work and work-to-home commutes.

## Conclusions

The current study is the first to examine the relationship between BMI and food environments around residential neighborhoods, work neighborhoods, and the commuting corridor. Significant results were found between BMI and the availability of food stores around residential neighborhoods and the commuting corridor, adjusted for individual-level factors. This study expands the analysis beyond residential neighborhoods, illustrating the importance of multiple environmental factors in relation to BMI.

## Supporting information

S1 File Multiple food environments and BMI dataset. (DTA)
